# Supplementary material for: γδ T Are Significantly Impacted by CLL Burden but Only Mildly Influenced by M-MDSCs
Source: Cancers (Basel). 2025 Jan 14;17(2):254. doi: 10.3390/cancers17020254 (PMC11763719; doi:10.3390/cancers17020254)
Supplement: Supplementary file 1 [file cancers-17-00254-s001.zip › sfig1-3.pdf]

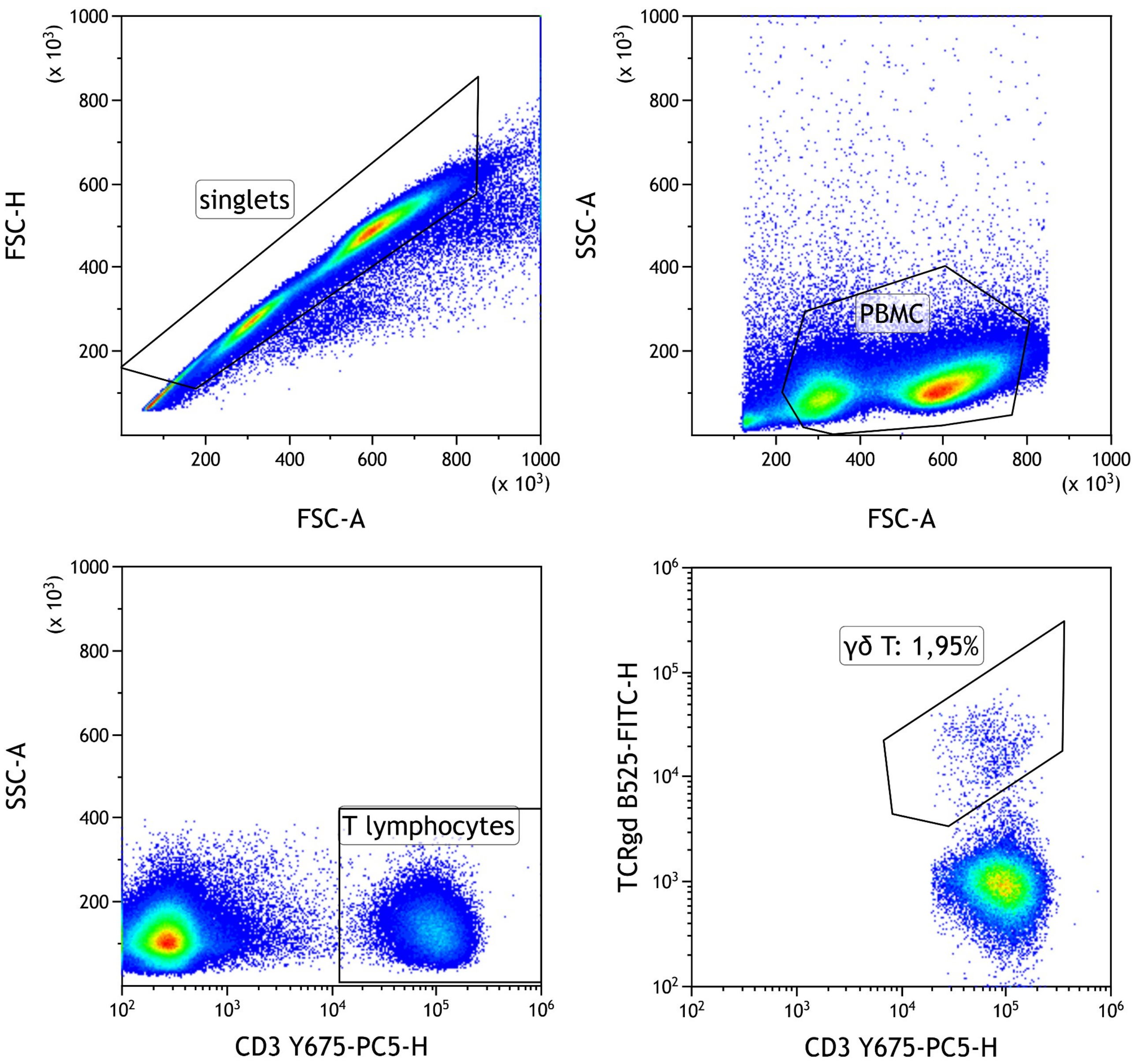

Supplementary Fig 1. γδ T gating strategy. At first, singlets were gated on FSC-Area vs FSC-height plot. Next, peripheral blood mononuclear cells were gated on FSC vs SSC, among those T lymphocytes were gated as CD3+. Finally, γδ T cells were gated among total T cells as TCRγδ+ cells.

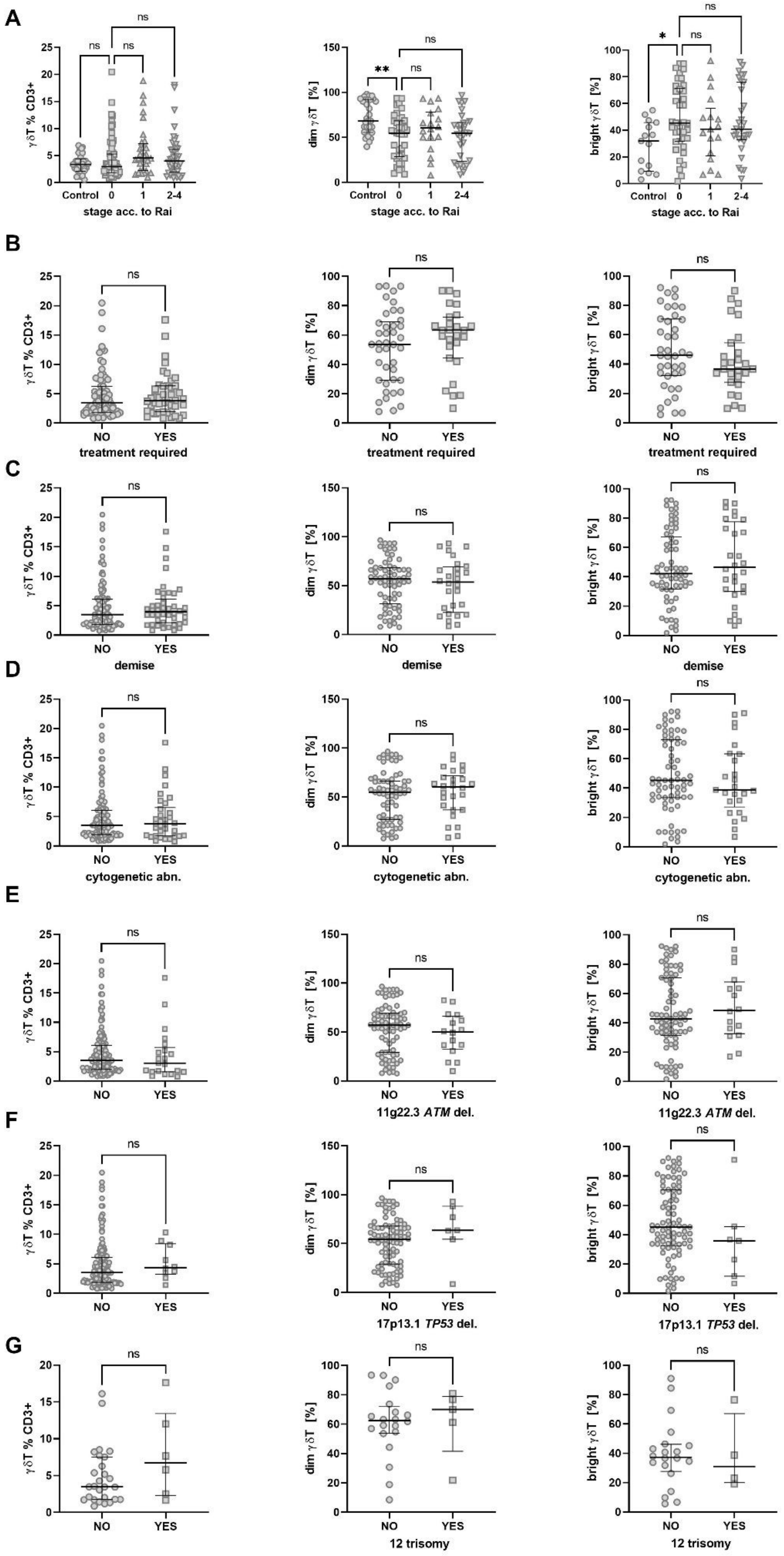

Supplementary Fig. 2. Analysis of total γδ T and their dim and bright subsets among subgroups of patients divided according to: Rai stage [**A**], whether treatment was required during follow-up [**B**], survival till the end of the follow-up [**C**], cytogenetic factors in general [**D**], *ATM* deletion [**E**], *TP53* deletion [**F**] and chromosome 12 trisomy [**G**].

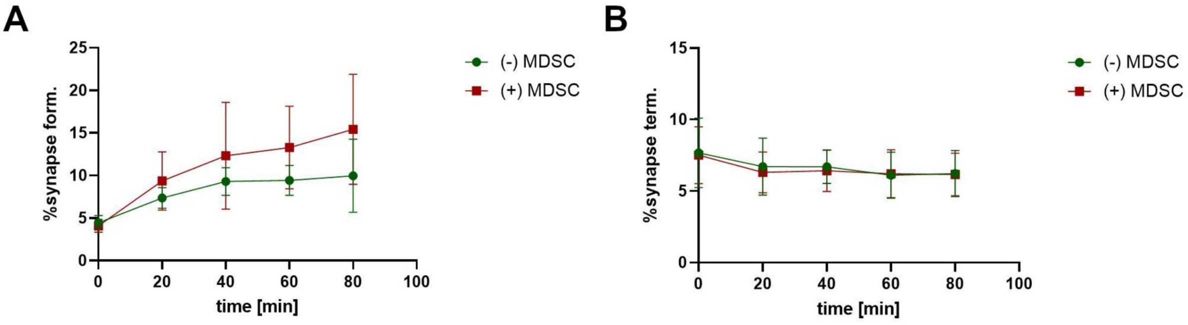

Supplementary Fig. 3. Synapse formation and stability. γδ T and γδ T exposed to M-MDSCs were co-incubated with Duller cells for 0 to 100min and the amount of Duller:γδ T aggregates was assessed by flow cytometry to study cytotoxic synapse formation [A]. Synapse stability (termination) was assessed by first incubating cells for 60 minutes and then shaking them for 0-100 minutes and measuring as previously [B]. No significant differences were observed.
