# Supplementary material for: γδ T Are Significantly Impacted by CLL Burden but Only Mildly Influenced by M-MDSCs
Source: Cancers (Basel). 2025 Jan 14;17(2):254. doi: 10.3390/cancers17020254 (PMC11763719; doi:10.3390/cancers17020254)
Supplement: Supplementary file 1 [file cancers-17-00254-s001.zip › sfig4-6.pdf]

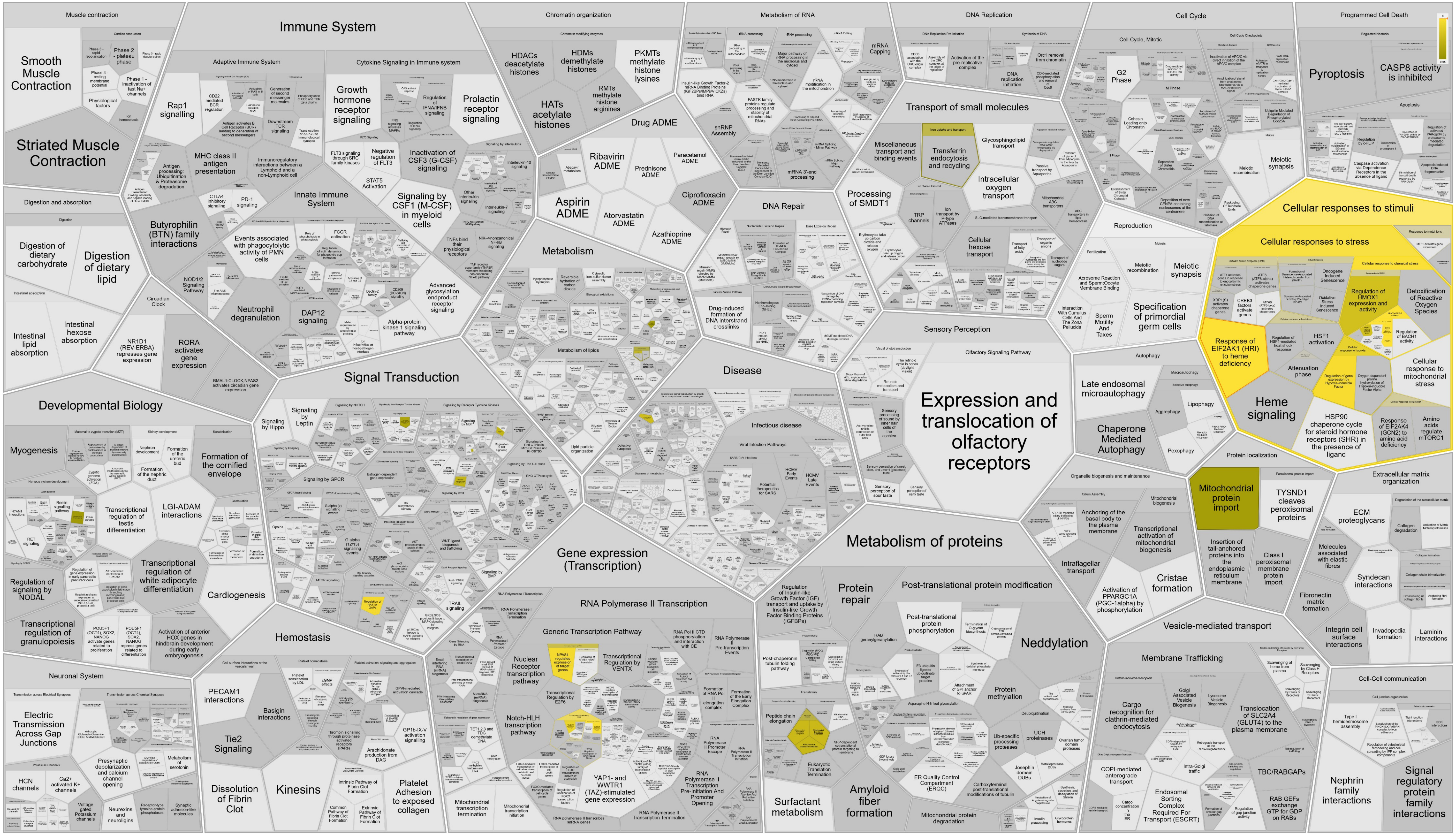

Supplementary Fig. 4. An overview of the pathways regulated in γδ T by M-MDSC exposure; generated from RNAseq data in reactome.org

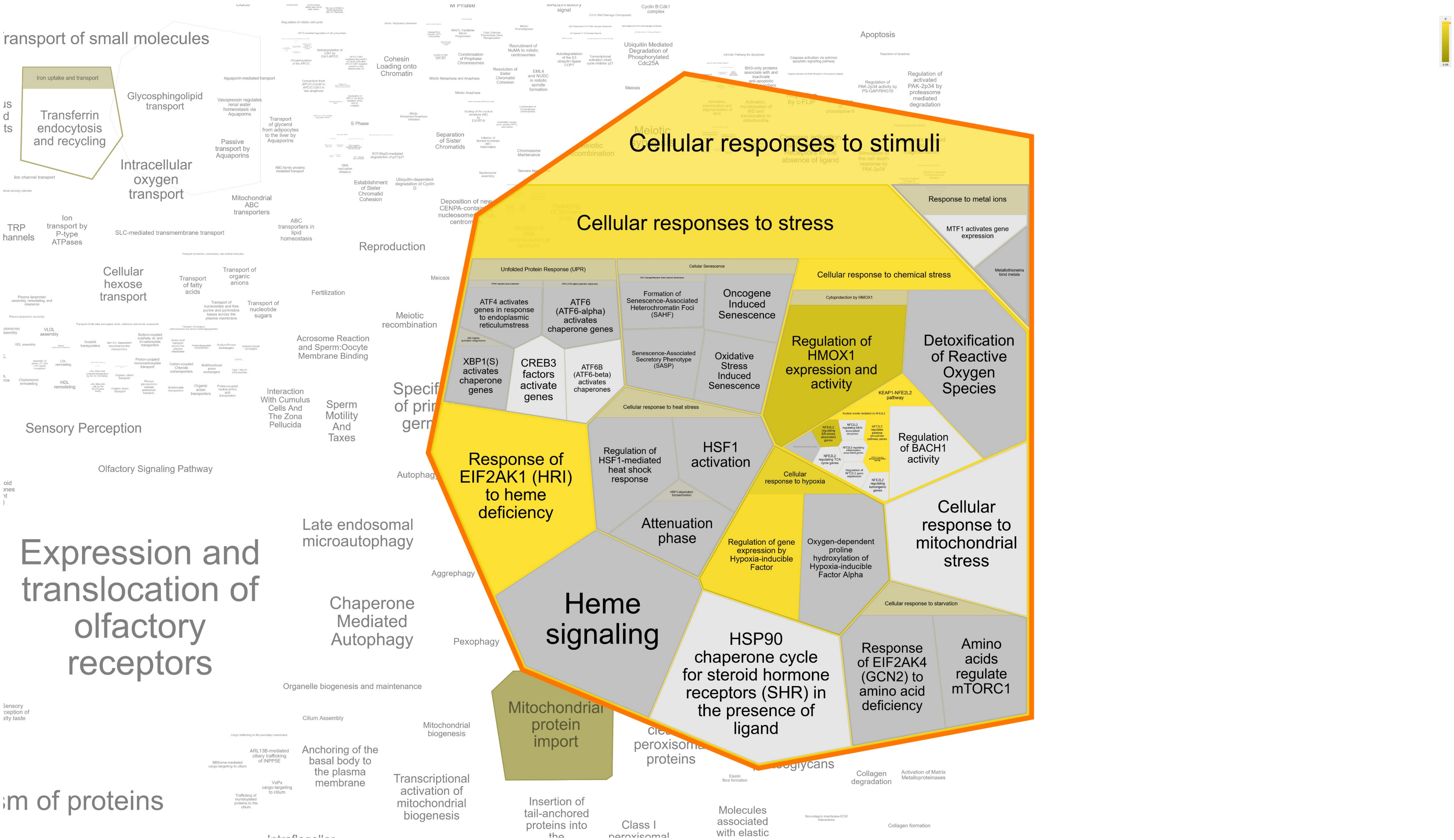

Supplementary Fig. 5. An overview of the subpathways related to TP53 and regulated in γδ T by M-MDSC exposure; generated from RNAseq data in reactome.org

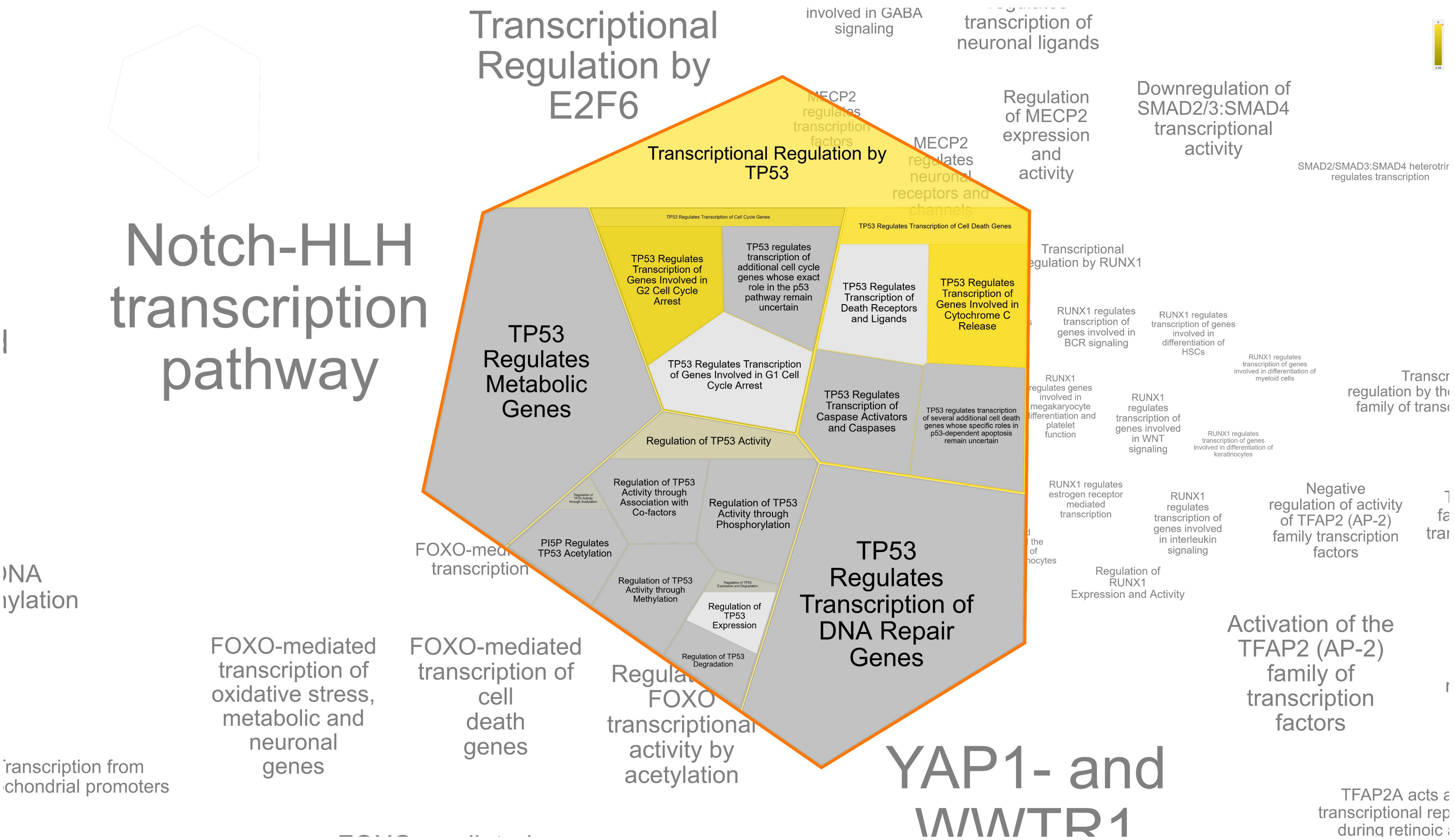

Supplementary Fig. 6. An overview of the subpathways of cellular response to stimuli regulated in γδ T by M-MDSC exposure; generated from RNAseq data in reactome.org
