# Supplementary material for: γδ T Are Significantly Impacted by CLL Burden but Only Mildly Influenced by M-MDSCs
Source: Cancers (Basel). 2025 Jan 14;17(2):254. doi: 10.3390/cancers17020254 (PMC11763719; doi:10.3390/cancers17020254)
Supplement: Supplementary file 1 [file cancers-17-00254-s001.zip › supp-table1.pdf]

Supp. Table 1. Sociodemographical characteristics of patients and healthy controls

	CLL patients	HV
Sex **	F: 49.69% (N=81)<br>M: 50.31% (N=82)	F: 50% (N=17)<br>M: 50% (N=17)
Age [Mean±SD] *	65.64±10.14	60.5±13.58
Treatment needed	No: 63.1% (N=82)<br>Yes: 36.9% (N=48)	
Demise during observation	No: 29.4% (N=48)<br>Yes: 70.6% (N=115)	
Rai stadium	0: 44.8% (N=69)<br>1: 21.4% (N=33)<br>2: 20.1% (N=31)<br>3: 7.8% (N=12)<br>4: 5.8% (N=9)	
Zap-70	low: 71.1% (N=113)<br>high: 28.9% (N=46)	
CD38	low: 63.5% (N=101)<br>high: 36.5% (N=58)	
IGVH	unmutated: 46% (N=29)<br>mutated: 54% (N=34)	
Cytogenetical abnormalities	No: 77.6% (N=125)<br>Yes: 22.4% (N=36)	

The cut-off point for low/hi CD38: 30%

The cut-off point for low/hi ZAP-70: 20%

* Mann Whitney U test: 0.076

** χ^2 test: 0.974
